# Early-Onset Inherited Metabolic Diseases: When Clinical Symptoms Precede Newborn Screening—Insights from Emilia-Romagna (Italy)

**DOI:** 10.3390/children12040464

**Published:** 2025-04-04

**Authors:** Giulia Montanari, Egidio Candela, Federico Baronio, Vittorio Ferrari, Giacomo Biasucci, Marcello Lanari, Rita Ortolano

**Affiliations:** 1 Specialty School of Pediatrics, Alma Mater Studiorum, University of Bologna, 40126 Bologna, Italy; giulia.montanari22@studio.unibo.it (G.M.); vittorio.ferrari4@studio.unibo.it (V.F.); 2 Pediatric Unit, IRCCS Azienda Ospedaliero-Universitaria di Bologna, 40138 Bologna, Italy; marcello.lanari@unibo.it (M.L.); rita.ortolano@aosp.bo.it (R.O.); 3 Department of Medical and Surgical Sciences, Alma Mater Studiorum, University of Bologna, Via Massarenti 11, 40126 Bologna, Italy; 4 Pediatrics and Neonatology Unit, Guglielmo da Saliceto Hospital, 29121 Piacenza, Italy; giacomo.biasucci@unipr.it; 5 Department of Medicine and Surgery, University of Parma, 43126 Parma, Italy

**Keywords:** expanded newborn screening, early-onset metabolic disorders, timing of screening, urea cycle disorders, organic acidemias, fatty acid oxidation disorders, methylmalonic acidemia, medium-chain Acyl-CoA dehydrogenase deficiency, Citrullinemia Type I, Argininosuccinic Acidemia

## Abstract

Background: Expanded Newborn Screening (ENS) allows the early identification of many inherited metabolic diseases (IMDs) for which timely treatment can modify the natural history. For most IMDs, diagnosis by ENS is pre-clinical. However, clinical symptoms may emerge for certain conditions before screening results become available. Methods: We describe six cases of patients with early-onset IMDs born between 2013 and 2023, who were admitted or transferred to Sant’Orsola University Hospital in Bologna (Italy). Results: Over the study period, 379,013 newborns underwent ENS in the Italian region of Emilia-Romagna. Excluding cases of congenital hypothyroidism, pre-clinical diagnoses from ENS were 410. In addition, six cases of IMD presented with early-onset clinical symptomatology, an antecedent to the outcome of newborn screening (incidence over 11 years of 1.58 cases per 100,000 infants). Among these patients, three were diagnosed with Urea Cycle Disorders (UCDs)—two with Citrullinemia type I (CIT1) and one with Argininosuccinic Acidemia (ASA); two were diagnosed with Methylmalonic Acidemia (MMA); and one was found to have Medium-Chain Acyl-CoA Dehydrogenase Deficiency (MCADD). Conclusions: Our 11-year experience with ENS has shown that clinical onset can occur between the second and fourth day of life, though rare. Even if dried blood spot (DBS) collection was performed 24–48 h after birth, the time required for sample transportation and processing would still delay result availability, making early intervention unlikely. Therefore, our experience supports performing ENS at 48–72 h, as currently implemented in Italy, while also highlighting the advantages and limitations of earlier screening.

## 1. Introduction

Expanded newborn screening (ENS) is an essential public health tool for secondary prevention that has changed the diagnostic landscape and natural history of inherited metabolic disorders (IMDs). Indeed, this system has allowed us to diagnose and start treatment as early as possible.

Diagnosis via ENS usually occurs before the onset of symptoms. However, certain metabolic disorders manifest clinically before screening results are available, posing a significant challenge for early identification and management in neonates.

Newborn screening is a cornerstone of modern public health, officially mandated in Italy since 1992. In the early years, newborn screening searched for only three pathological conditions. In contrast, over the years, it has expanded significantly, mainly due to the advent of tandem mass spectrometry (MS/MS) and the expansion of the panel through complete nationwide coverage under Law 167/2016 to search for 49 pathologies. The Ministry of Health set up a periodic review of the list of diseases screened for [1].

To optimize accessibility and diagnostic efficiency, Italy consolidated its screening centers from 32 to 15, ensuring a streamlined approach. The gradual introduction of different second-tier tests (2-TT), performed on the same initial Dried Blood Spot (DBS) sample, allows a lower threshold of false positives and a decrease in families’ stress.

Most countries, including Italy, recommend collecting DBS samples between 48 and 72 h after birth. Research has shown that collecting samples too early (before 48 h of life) may lead to unreliable results due to physiological fluctuations in metabolic markers during the transition from intrauterine to extrauterine life [2,3,4]. For instance, thyrotropin (TSH) undergoes a natural surge postnatally, which can result in false positives for congenital hypothyroidism if measured too soon. Similarly, amino acids such as phenylalanine and tyrosine exhibit considerable variability within the first hours of life, especially in neonates born to mothers with gestational diabetes mellitus (GDM) [5,6].

Another critical limitation of early sampling is the potential for false negatives. Some metabolic disorders, particularly those affecting fatty acid oxidation, require more time to become biochemically detectable. Their hallmark metabolic abnormalities (such as elevated acylcarnitines) only emerge after fasting or sustained milk feeding. If screening is performed too early, the necessary metabolic transitions may not have occurred, leading to missed diagnoses that could have been identified with later sampling [2,7].

At the same time, certain IMDs can present with life-threatening symptoms in the neonatal period, even before screening results are available. Organic acidurias (OAs) and urea cycle defects (UCDs) can cause rapid metabolic decompensation within the first days of life. Symptoms such as poor feeding, vomiting, lethargy, seizures, or respiratory distress should raise immediate suspicion of an underlying metabolic disorder, prompting urgent diagnostic testing and therapeutic intervention. This issue underscores the necessity for continuous awareness and specialized training among healthcare professionals, particularly in Maternity Wards and Neonatal Intensive Care Units (NICUs). Since newborn screening is not an immediate diagnostic tool but rather a population-based screening method, clinicians must remain vigilant for early clinical signs that may indicate an IMD before laboratory confirmation is available. In this case, empirical management (including metabolic stabilization, intravenous glucose administration, and dietary modifications) can be lifesaving in these cases. Therefore, continuous education and training are crucial for effectively managing IMDs.

A well-balanced protocol must be maintained to maximize the benefits of newborn screening. This agreement includes proper sample collection timing (ideally between 48 and 72 h), an efficient logistics system for sample transportation (six to seven days per week), and a screening laboratory equipped to analyze results and initiate follow-ups without delay.

While newborn screening remains a powerful tool in neonatal care, its limitations highlight the need for a proactive clinical approach. ENS’s main strength is diagnosing diseases without early clinical onset, such as aminoacidopathies [8], or diseases in which clinical onset is in the first days of life but usually later than the first week, as in congenital adrenal hyperplasia [9]. Healthcare providers must recognize that some inherited metabolic disorders can present before screening results are available, emphasizing the importance of early clinical suspicion (Table 1).

This article will present six cases of IMDs managed at the Inherited Metabolic Diseases Unit of Sant’Orsola Hospital in Bologna. We will describe the types of clinical manifestations observed and outline the diagnostic and therapeutic approach taken in each case.

## 2. Materials and Methods

This study is a single-center, retrospective analysis conducted at the Regional Reference Clinical Center for Newborn Screening of Endocrine and Metabolic Diseases in Bologna. We reviewed data from the ENS program in Emilia-Romagna over an 11-year period (2013–2023), identifying all cases of early-onset IMDs diagnosed within this timeframe.

The inclusion criteria were as follows: (1) newborns who underwent ENS; (2) clinical onset within the first five days of life; (3) admitted or transferred to our institution to receive clinical care; and (4) confirmed IMD diagnosis. The diagnosis was initially suspected based on ENS data and subsequently confirmed by molecular analysis in all cases. Due to the retrospective design of the study, case identification was limited to clinically evident presentations that were documented in medical records; therefore, the possibility remains that some very early-onset cases of metabolic disease went unrecognized.

For incidence calculations, we utilized data from the annual summary document (2013–2023) created by a group of experts to collect data about the screening activity of the Italian Society for the Study of Inherited Metabolic Diseases and Newborn Screening (SIMMESN) [10]. Clinical data were retrospectively analyzed to assess the role of newborn screening in disease management, diagnostic accuracy, and therapeutic decision making.

In our region, Emilia-Romagna (Italy), ENS for inherited metabolic diseases was implemented in 2011 as a simple pilot project in the early years. DBS samples are collected between 48 and 72 h of life. A dedicated transport system ensures the timely delivery of samples from birth centers to a single regional laboratory, where all DBS analyses are performed, and any abnormal findings are promptly reported to a centralized clinical center. The entire data transmission system is digitalized.

## 3. Results

Between 2013 and 2023, 379,013 newborns underwent screening in the Italian region of Emilia-Romagna. Excluding cases of congenital hypothyroidism, 410 diagnoses (incidence of 108 per 100,000 infants) of endocrine-metabolic diseases were made pre-clinically through ENS during the study period.

Six cases of IMDs with clinical onset before screening results were identified, with an incidence of onset of around 1.58 cases per 100,000 infants over 11 years (Table 2):

- Three cases of urea cycle disorders (two Citrullinemia Type I [CIT1] and one Argininosuccinic Acidemia [ASA]).
- Two cases of Methylmalonic Acidemia (MMA).
- One case of Medium-Chain Acyl-CoA Dehydrogenase Deficiency (MCADD), a fatty acid oxidation disorder.

### 3.1. Case 1

A male infant was born at 38 + 1 weeks via spontaneous vaginal delivery after a pregnancy complicated by gestational diabetes and pre-eclampsia. The parents were consanguineous (first cousins). The newborn had a birth weight of 3560 g (79th percentile), with Apgar scores of 9 at 1 min and 10 at 5 min of life.

On the second day of life, he was admitted to the Neonatal ICU due to tachypnea, lethargy, and tremors. Because of a rapidly increasing hyperammonemia (peak: 770 µmol/L), total parenteral nutrition (TPN) was initiated, along with intravenous (IV) detoxification therapy, and a Tenckhoff catheter was placed for peritoneal dialysis. Ammonia levels progressively normalized, allowing the discontinuation of dialysis on day 7. On day 4, he developed seizures with corresponding electroencephalographic abnormalities, prompting the initiation of phenobarbital treatment. Brain MRI revealed diffuse white matter signal abnormalities and thinning of the corpus callosum. Currently, at six and a half years old, he has residual epileptic encephalopathy and remains on anticonvulsant therapy.

Newborn screening performed at 48 h of life revealed markedly elevated citrulline levels associated with low arginine, confirmed by plasma amino acid analysis. The screening result was available on the fourth day of life, allowing for timely adjustment of the subsequent therapeutic approach. The diagnosis of citrullinemia type 1 was genetically confirmed, with a homozygous genotype for the c.1168G > A mutation in the *ASS* gene.

### 3.2. Case 2

A female patient was born at 37 weeks of gestation via eutocic delivery following an uneventful pregnancy, although prenatal ultrasound had raised suspicion of right kidney atrophy. Adaptation to extrauterine life was normal.

At 48 h of life, she developed tachypnea, accompanied by weight loss greater than 10%, leading to admission to the NICU. Blood tests revealed metabolic acidosis and hyperammonemia (peak: 1218 µmol/L). Consequently, she was placed on fasting and started on IV glucose infusion. Detoxification therapy with sodium benzoate and arginine was initiated as an emergency measure, followed by maintenance treatment. Due to persistently elevated ammonia levels, a second detoxification protocol was administered, and peritoneal dialysis was initiated. Cranial ultrasounds revealed marked periventricular white matter hyperechogenicity, while an abdominal ultrasound showed hepatomegaly with mild hyperechogenicity.

Newborn screening was performed at 48 h of life. The results came on the fourth day of life and reported markedly elevated citrulline levels (1038 µmol/L; normal range: 4–90 µmol/L). The screening-based suspicion of Citrullinemia Type 1 (CIT1) was subsequently confirmed by molecular analysis of the *ASS* gene (heterozygous genotype carrying c.905T > G and c.1030C > T mutations).

Despite intensive treatment, at two weeks of life, the infant developed severe hypotension and refractory cardiocirculatory failure, unresponsive to high-dose vasopressors, progressing to multi-organ failure and exitus on the 19th day of life.

### 3.3. Case 3

A male twin was born at 36 + 1 weeks via urgent cesarean section for maternal pre-eclampsia, initially stable but developing lethargy and anuria on day 4, followed by worsening hypotonia and hyperammonemia (peak: 1381 µmol/L) on day 5.

Therapy included IV sodium benzoate and arginine with an initial double-loading dose followed by continuous infusion and 10% dextrose to provide an alternative energy source. Additional supportive therapy included IV carnitine and intramuscular vitamin B12. Peritoneal dialysis was initiated via a surgically placed Tenckhoff catheter on day 5 to reduce ammonia levels rapidly.

Before clinical onset, the DBS sample had already been collected between 48 and 72 h of life, before clinical onset. Therefore, the laboratory was notified of the urgency of the analysis request. The results of ENS came on the fifth day of life and revealed significantly elevated methionine (60.77 µmol/L; normal < 45 µmol/L) and citrulline (289.77 µmol/L; normal < 90 µmol/L), with subsequent re-testing confirming further increases in methionine (83.39 µmol/L), citrulline (500.30 µmol/L), and alanine (749.61 µmol/L; normal < 600 µmol/L). Plasma amino acid analysis showed a marked accumulation of argininosuccinic acid (2758 µmol/L). The diagnosis of argininosuccinic aciduria was confirmed via molecular genetic testing (heterozygous genotype, with mutations c.1129C > G and c.1322G > A in the *ASL* gene).

Cranial ultrasound revealed periventricular white matter hyperechogenicity with heterogeneous frontal involvement and mild basal ganglia hyperechogenicity. Abdominal ultrasound showed hepatomegaly with markedly increased echogenicity.

The initial improvement led to the discontinuation of dialysis on day 7. Still, the patient subsequently deteriorated with metabolic acidosis, severe hypotension, unresponsiveness to fluid therapy, hyperlactatemia, and hyperglycemia despite normal ammonia levels. Despite intensive metabolic and supportive treatment, he developed multi-organ failure with exitus on day 11.

### 3.4. Case 4

A male was born at 41 + 1 weeks of gestation via spontaneous delivery following a pregnancy complicated by maternal hypothyroidism. Perinatal adaptation was unremarkable, and the first day of life proceeded usually, with good tolerance to breastfeeding. On the second day of life, there was only a slight reduction in feeding. However, on the third day, the infant developed tachypnea, reduced responsiveness, and hypotonia. He was subsequently transferred to the NICU, where blood tests revealed metabolic acidosis and hyperammonemia (peak: 849 µmol/L). Feeding was discontinued, and IV glucose infusion was immediately initiated, along with detoxification therapy using arginine, carglumic acid, and sodium benzoate. Additionally, carnitine and hydroxocobalamin supplementation was initiated.

He was then transferred to the Pediatric Intensive Care Unit (PICU), where Continuous Veno-Venous Hemofiltration (CVVH) was started. Dialysis was discontinued on the sixth day of life following normalization of blood ammonia levels. Due to the presence of neutropenia, anemia, and thrombocytopenia, the patient required plasma and red blood cell transfusions.

ENS results were available on the fourth day of life and raised suspicion of methylmalonic aciduria due to elevated levels of methylmalonic acid and propionyl carnitine (C3: 33.69 µmol/L; cut-off value: <0.40 µmol/L) detected upon DBS analysis. Urinary organic acid testing confirmed increased methylmalonic acid levels (2112 mM/MCREU; cut-off value: <2 mM/MCREU), and the diagnosis was further confirmed by molecular analysis of the *MUT* gene (mutations c.785G > A/c.2104_2197delGCCGinsTGGAA, related to MMA_mut0).

Following hospital discharge, the child has continued dietary and pharmacological therapy and has subsequently undergone liver transplantation.

### 3.5. Case 5

A male patient was born at term following an uneventful pregnancy. On the third day of life, he was re-admitted to the hospital due to lethargy approximately 12 h after being discharged home. Upon admission, he presented with severe metabolic acidosis and hypovolemic shock.

Blood tests revealed hyperammonemia (peak: 1100 µmol/L). He was immediately started on IV detoxification therapy and underwent peritoneal dialysis, which was continued until the sixth day of life. Additionally, treatment with hydroxocobalamin, thiamine, and carnitine was initiated.

Newborn screening results came on the fourth day of life. They revealed markedly elevated levels of methylmalonic acid and propionyl carnitine (C3: 22.34 µmol/L) on DBS analysis, which were further confirmed by urinary organic acid quantification (methylmalonic acid: 1212 mM/MCREU), leading to a suspected diagnosis of methylmalonic aciduria. Genetic testing definitively confirmed the diagnosis (homozygous genotype for the c.1207C > T mutation in the *MUT* gene, associated with MMA_mut0).

Due to the suspicion of critical events, an electroencephalogram (EEG) was performed, which ruled out epileptic activity but confirmed findings consistent with severe metabolic encephalopathy. Serial cranial ultrasound monitoring revealed increased echogenicity of the basal ganglia.

Currently, at 11 years of age, the patient has undergone liver transplantation following years of pharmacological and dietary therapy, with regular follow-up at our center.

### 3.6. Case 6

A female infant was born at term from consanguineous parents following a pregnancy complicated by gestational diabetes and maternal hypertension. After an uneventful adaptation to extrauterine life, at 48 h of life, she developed hypotonia, hypothermia, and fasting-induced hypoglycemia associated with metabolic acidosis. IV infusion of glucose and bicarbonate was immediately initiated; however, the clinical course was further complicated by severe heart failure, requiring boluses of epinephrine and urgent transfer to the Pediatric Cardiology Intensive Care Unit for continued inotropic support.

DBS sample collection was performed at 48 h of life, and the ENS results, which were available at 72 h of life, raised suspicion of MCADD based on the detection of an acylcarnitine profile compatible with the disease, with elevated C8 and C6 and low free carnitine levels. The diagnosis was confirmed by measuring residual *ACADM* enzymatic activity (0%), and molecular genetic analysis (homozygous genotype for the mutation c.997A > G in *ACADM* gene). Abdominal ultrasound revealed a mildly enlarged and hyperechoic liver. On the fifth day of life, high-flow IV dextrose infusion was initiated, along with oral carnitine supplementation and the introduction of a formula with low-content medium-chain lipids (MCTs).

The infant has started weaning with good tolerance, maintaining fasting intervals of approximately 4 h during the day and 4–5 h at night. An emergency diet, including maltodextrin supplementation, is implemented in the case of feeding refusal.

## 4. Discussion

In our experience, the incidence of early neonatal acute metabolic decompensation is about 1.58 cases per 100,000 infants over 11 years, a much lower value compared to the incidence of diagnosed hereditary metabolic disorders. These initial data allow us to state that this clinical manifestation is an event that can be defined as very rare, although not negligible. The diagnosed conditions described (2 CIT1; 1 ASA; 2 MMA_ Mut0 and 1 MCADD) are among the conditions at risk for early acute decompensation (UCDs, OAs, and FAODs), as reported in Table 1. The presented symptomatology, characterized by symptoms secondary to hyperammonemia or hypoglycemia, represents the classic manifestation of acute metabolic decompensation. Therefore, it is always crucial for clinicians working in birth centers to be well-educated and continuously trained in recognizing and managing these conditions.

The greater difficulty in treating hyperammonemia in cases 1, 2, 3, and 5, compared to patient 4—the most recently diagnosed and the only survivor without sequelae—could, in a preliminary analysis, be attributed, among various factors, to the use of peritoneal dialysis rather than CVVH. In fact, current UCD guidelines recommend the immediate initiation of extracorporeal dialysis if plasma ammonium concentration exceeds 500 μmol/L or if it is above 250 μmol/L without an adequate decrease within 3 to 6 h after the start of metabolic emergency treatment [11,12]. Based on our limited experience, it appears that this reasoning may apply not only to urea cycle disorders but also to organic acidurias.

The 10 criteria for population-based screening of disorders established by Wilson and Junger in 1968 [13] and later revised and expanded by the World Health Organization (WHO) in 2008 and 2011 [14] have become and remain the gold standard for public health screening policies. Despite not being initially intended for this purpose, these criteria are a framework for evaluating which disorders should be incorporated into ENS panels.

In 1998, the UK National Screening Committee (UKNSC) established 17 criteria for evaluating the feasibility and effectiveness of screening programs [15]. Both these and the Wilson and Jungner criteria suggest that a disorder should have a latent or pre-symptomatic phase to justify screening, assuming symptoms will naturally lead to a diagnosis. However, inherited metabolic diseases often present nonspecific symptoms, frequently mistaken for more common conditions, causing significant diagnostic delays in newborns. Therefore, early symptomatic onset should not exclude a disease from neonatal screening.

Currently, the Recommended Uniform Screening Panel (RUSP) for Newborn Screening in the United States includes at least 20 inherited metabolic disorders among the ‘Core Conditions’ recommended for ENS and an additional 22 metabolic disorders among the ‘Secondary Conditions’. Notably, all disorders described in our case series—methylmalonic acidemia, citrullinemia type 1, medium-chain acyl-CoA dehydrogenase deficiency, and argininosuccinic aciduria—are included in the ‘Core Conditions’ list [16]. Given these disorders’ severity and potential treatability, screening is considered essential.

In the USA, newborn screening programs are required to report positive results for critical conditions to the clinician by the fifth day of life, necessitating specimen collection before 48 h of life and prompting many programs to adjust their business and holiday hours to comply with this recommendation. From 2016 to 2018, the percentage of specimens collected within 48 h of life increased from 95% to 97% in 25 states, with 80% of programs achieving a collection rate of specimens within this timeframe above 90% [17]. However, while analytic cutoffs are based on normal ranges for infants 24–48 h old, earlier testing may impact analytical validity and accuracy, increasing both the number of specimens flagged for follow-up and the risk of false negatives due to metabolic immaturity and transient biochemical fluctuations in neonates.

### 4.1. Impact of Expanded Newborn Screening on Early-Onset Metabolic Disorders

Studies have consistently demonstrated the effectiveness of Expanded Newborn Screening in detecting metabolic disorders at an early stage. Wilcken et al. (2009) analyzed outcomes in screening for organic acidurias, urea cycle defects, and fatty acid oxidation disorders, highlighting that ENS doubled the detection rate compared to clinical diagnosis alone [18]. Although early presenting cases of OAs and UCDs often had severe outcomes (high morbidity and mortality) despite screening, ENS significantly improved the prognosis for later-onset cases [19]. Notably, screening for MCADD prevented both metabolic crises and death, confirming the value of early detection. The study supports ENS at 48–72 h, which enables rapid biochemical diagnosis and timely intervention, particularly for FOADs and later-presenting metabolic conditions. A notable finding is the high mortality (about 50%) among early-presenting cases (≤5 days, 16% of diagnoses), which could mean that this type of patient has little benefit from pre-symptomatic screening. Nonetheless, ENS often played a central role in these patients as it allowed for quicker diagnosis, reduced the material time of prescribing specialist examination and specimen collection, and helped clinical management. In some cases, it also provided a post-mortem diagnosis that could help families through the process of genetic counseling [18,19,20]. These findings highlight both the clinical and the ethical dimensions of early-onset IMD. From a clinical point of view, ENS, although it cannot always change the outcome of severe neonatal presentations, may facilitate early diagnosis, especially when life-threatening symptoms occur before results are available. From an ethical point of view, ENS, if accompanied by adequate informed parental consent, may be useful in informing their reproductive choices.

In another work, Heather et al. (2024) were concerned about ambiguity and a lack of uniformity in disorder counting on newborn screening panels between Australia, New Zealand, and California [21]. Their study highlights the importance of decision-making considering the healthcare system’s ability to manage true positives and promptly identify false positives or uncertain cases. In rare conditions, patients may require years of follow-up before a definitive diagnosis is reached [22]. Cost-effective and socially acceptable interventions should remain a key ethical and policy principle.

Reflecting this principle, the Human Genetics Society of Australasia (HGSA) Newborn Screening committee has excluded ornithine transcarbamylase (OTC) deficiency and non-ketotic hyperglycinemia (NKH) from their screening panel, primarily based on two key observations: the early onset of symptoms relative to the neonatal screening result and the lack of reliable laboratory markers.

These two different studies offer the opportunity to provide at least two types of perspectives: while the effectiveness of early therapeutic intervention remains the primary goal, the diagnostic usefulness of screening remains critical for both families and clinicians.

Adding further complexity, the long-term clinical impact of ENS remains uncertain. A study by Grünert et al. [23] assessing propionic acidemia (PA) patients during follow-up found that while early diagnosis through newborn screening was associated with a lower mortality rate, no significant benefit was observed in surviving patients regarding their clinical course, using the following as parameters: the number of metabolic crises, neurocognitive and physical development, and long-term complications. Therefore, the main impact of newborn screening appears to be on mortality, although it does not permanently alter the overall disease trajectory for some metabolic disorders.

For early-onset IMDs, which often escape the traditional timing of ENS, the latest advances in point-of-care testing and rapid molecular diagnostics could make a difference, especially in NICUs as tools to complement ENS [24,25]. Ultra-rapid whole genome sequencing has shown promising diagnostic turnaround times (less than 24 h), allowing timely interventions in high-risk newborns admitted to NICUs. However, the evolution of diagnostic technologies toward rapid molecular tests or even genome-based approaches dictates the need to review current informed consent policies to ensure transparency, parental autonomy, and alignment with public health strategies [26].

### 4.2. Challenges and Considerations in the Timing of Screening

Several long-term studies conducted in different nations have analyzed this issue, reporting a percentage of early onset cases—before the availability of screening results—ranging from 2.9% to 17%, with a DBS collection time ranging from 24 h to 7 days of life. In most cases, the patients suffered from PA, CIT1, MMA, MCADD, and maple syrup urine disease (MSUD) [18,20,27,28,29,30,31,32].

For example, a long-term study in Germany [28], which followed 306 patients recalled at newborn screening for pathological findings over a median of 6.2 years, emphasized that the effectiveness of ENS relies not only on the timing of sampling but also on other links of the chain. In detail, the study identified transport inefficiencies as the weakest link in the system while improving sample collection and laboratory analysis. Another interesting finding is that in the 28 patients with early onset compared to the closing times of neonatal screening, none had a metabolic decompensation following the DBS outcome, thus further underlining the importance of timing.

Similarly, a Galician study [30], which analyzed 440.723 newborns screened over 22 years, found that in 4% (n = 13) of patients, clinical signs of metabolic intoxication emerged before ENS results were available (at a median age of 3 days), including cases of citrullinemia type 1 (CIT1), maple syrup urine disease (MSUD), propionic acidemia (PA), methylmalonic acidemia (MMA), and carnitine-acylcarnitine translocase deficiency (CACTD). Analyzing the work in detail, a significant difference in effectiveness can be seen between the period before 2003, when samples were collected later (between the fifth and eighth day after birth), in which 7 cases of acute neonatal decompensation occurred, and the subsequent period, in which the change in collection policy led to an advance to 24–72 h of life and a significant improvement. In addition, the system was further optimized by working on the transport system and ensuring faster delivery.

Another example is the Portuguese Neonatal Screening Program (PNSP), which screened over 1.7 million neonates between 2004 and 2022, adopting a sample collection window of 3–6 days after birth, with an average referral age for positive cases at 10.1 days. This timing allows for more reliable biochemical assessments, although it is more detrimental to the ability to intercept early onset. The Portuguese experience remains in line with other screening programs [33].

Lastly, a population-based study analyzing data from the California Newborn Screening program found that early specimen collection (≤24 h) was associated with elevated marker levels for conditions like phenylketonuria (PKU), isovaleric acidemia (IVA), and MMA, leading to a higher false-positive rate. Conversely, later collection (≥49 h) resulted in decreased metabolite levels, increasing the likelihood of false negatives, particularly for disorders such as OTC deficiency [34].

The proposal to bring forward neonatal screening to 24–48 h after birth, instead of the current window of 48–72 h, is a change that, although intuitively, could seem to facilitate early diagnosis of the most serious forms, in practice lends itself to significant challenges, which can be summarized in three main points:

- Logistics issues, with sample transportation and analysis delays, could still prevent timely admission.
- Newborns at 24–48 h have not yet ingested sufficient milk, potentially leading to false negatives for disorders requiring metabolic stress (e.g., some aminoacidopathies, organic acidurias).
- Early collection increases the likelihood of inconclusive or ambiguous results, causing an increase in false positives and, therefore, impacting the social and family costs of unnecessary follow-up.

Crucially, despite the many limitations due to its retrospective design (e.g., data collected from medical records may be subject to incomplete documentation or changes in clinical practice over time), our study can provide valuable insights into the rare but significant cases of early symptomatic presentation, prior to the availability of ENS results. Equally important is how this opens a broader discussion on the modalities used by different screening programs around the world: how the challenge regarding timing is addressed, particularly about the time window between DBS collection and laboratory reporting of results, and how this can be adjusted.

Considering our experience, we observed that clinical onset occurred between the second and fourth day of life in all six cases. Therefore, even if DBS collection were anticipated to be 24–48 h after birth, it would not have prevented the onset of symptoms, as the time required for sample transportation and processing would still delay the availability of results. Overall, while improved logistics can undoubtedly be of great help, this advance alone cannot necessarily positively impact and respond to the challenges mentioned above. Thus, our experience supports the 48–72-h ENS, which is currently in use in Italy.

## 5. Conclusions

In our cases, clinical symptoms preceded the newborn screening results. This observation raises the question of whether ENS is redundant for disorders with extremely early-onset presentation, leading to the perception that, in such cases, diagnosis should be frequently based solely on clinical evaluation and, in turn, raising ethical and economic concerns regarding the cost-effectiveness of screening for these conditions.

However, we argue that continuing to include these conditions in the ENS panel remains highly beneficial, as screening facilitates a more targeted therapeutic approach, ensuring optimal patient care while also helping to prevent potential clinical misdiagnosis, as initial symptoms may be highly nonspecific and overlap with other conditions. The two critical clinical conditions that must be recognized in newborns at birth are those associated with the symptomatology of hypoglycemia and hyperammonemia, both of which were also observed in the case series we reported. Another fundamental aspect is relief in managing a clinical condition as soon as it is known, both for clinicians and families who can see the challenge to face as quickly as possible.

Specifically, in the case of patient 4, who exhibited clinical onset on the third day of life, the availability of a dried blood spot (DBS) sample—although not yet analyzed—allowed for an urgent, rapid laboratory assessment. This significantly expedited the diagnosis of MMA_Mut0 and enabled the prompt initiation of a targeted therapy, which contributed to a favorable outcome for the patient.

Based on our experience, two additional prognostic factors may be critical: ensuring the availability of emergency detoxification drugs in all spoke-level birth centers and equipping hub centers with CVVH along with trained personnel capable of its effective use.

Further discussion, although separate, concerns diagnosis in late-onset phenotypes, which, in the absence of early symptoms, would certainly escape neonatal management and only come to attention when complications were often irreversibly present. This axiom is particularly relevant for urea cycle disorders (UCDs), as evidence suggests that approximately two-thirds of UCD patients remain asymptomatic until a median age of 12 days, the typical age at diagnosis for cases identified through ENS, highlighting the potential benefit of early detection in preventing metabolic crisis and improving long-term outcomes [35].

Notably, the timing of ENS alone cannot fully resolve the challenges posed by early metabolic decompensation. Although the latest advances point toward the increasing implementation of genetic newborn screening, a multidisciplinary approach —involving neonatologists, metabolic specialists, and genetic counselors—is essential from the very first hours of patient management, and the centralization of cases is fundamental for implementing the most effective therapeutic protocols and optimizing outcomes.

In a recent online Delphi study (2023) involving pediatricians and IMD experts conducted to refine the definition of treatability in the context of ENS, a treatability score was introduced to assess eligibility for screening. The study identified the statement “The expected benefit/burden ratio of early treatment is positive and results in a significant health benefit” as the most influential in decision making [36]. This aligns with our position that ENS performed between 48 and 72 h of life remains an effective strategy in cases of early metabolic crisis, allowing timely biochemical diagnosis and early therapeutic interventions in most cases.

The achievements and significant benefits of ENS should not, therefore, lower the guard of healthcare personnel employed in Birth Centers and Neonatal Intensive Care Units, where alertness and training must always be very high since it is there that the most crucial diagnostic challenge occurs.

Lastly, the early onset of these conditions highlights the importance of maintaining a high level of logistical and infrastructural organization of newborn screening. The collection of DBS samples between 48 and 72 h of life, ensuring a reliable 6-day-per-week transportation system, and maintaining a well-equipped, high-capacity Screening Laboratory is fundamental to maximizing the effectiveness of expanded newborn screening programs. In fact, in all the six cases presented, screening results were available between the fourth and fifth day of life, thanks to timely DBS collection and rapid analysis by the dedicated laboratory, still allowing for guidance in the subsequent therapeutic approach.

## Figures and Tables

**Table 1:** Main classes of IMDs with potential early onset: clinical and therapeutic characteristics.

Disease Category	Included Disorders	Clinical Onset	Laboratory Findings at Onset	Onset Timing	Therapeutic Management
Urea Cycle Disorders (UCDs)	- CPS1 Deficiency - OTC Deficiency - Citrullinemia Type 1 (ASS1) - Argininosuccinic Aciduria (ASL) - Hyperargininemia (ARG1)	Progressive lethargy Cyclic vomiting Tachypnea Seizures Hypotonia Hyperammonemic encephalopathy	- Severe hyperammonemia (>200–500 μmol/L) - Respiratory alkalosis (early stages) - Low plasma citrulline levels (CPS1 and OTC deficiencies) - Increased orotic aciduria (OTC deficiency)	24–72 h of life (severe CPS1 and OTC deficiencies); Milder forms may manifest later with episodic encephalopathy	- Protein intake restriction - Dialysis/hemodialysis - Sodium benzoate and phenylbutyrate - Arginine supplementation (except in ARG1 deficiency)
Organic Acidemias (OAs)	- Methylmalonic Acidemia (MMA) - Propionic Acidemia (PA) - Isovaleric Acidemia (IVA) - Glutaric Acidemia Type 1 (GA-1)	Persistent vomiting Lethargy, hypotonia Respiratory failure Seizures Metabolic encephalopathy	- Metabolic acidosis with increased anion gap; lactic acidosis; ketoacidosis - Moderate hyperammonemia (<200–300 μmol/L) - Elevated acylcarnitines (e.g., C3 in PA and MMA, C5 in IVA)	Typically between days 2–5 after the start of feeding	- Protein intake restriction - IV glucose hydration - Carnitine supplementation (MMA, IVA) - Hemodialysis (severe cases)
Fatty Acid Oxidation Disorders (FAODs)	- Medium-chain acyl-CoA dehydrogenase deficiency (MCAD) - Very-long-chain acyl-CoA dehydrogenase deficiency (VLCAD) - Long-chain 3-hydroxyacyl-CoA dehydrogenase deficiency (LCHAD) - Carnitine palmitoyltransferase I/II deficiency (CPT1/CPT2)	Hypoglycemia Lethargy, hypotonia Liver failure and cardiomyopathy (severe cases) Rhabdomyolysis (LCHAD/CPT2 defects)	- Hypoglycemia with low ketone levels - Altered plasma acylcarnitines (C8–C10 in MCAD; C14–C18 in VLCAD; C16–C18 in LCHAD/CPT2) - Elevated ammonia (severe cases)	Between days 2–7 after prolonged fasting or illness	- Immediate IV glucose - Avoid prolonged fasting and ensure a carbohydrate-rich diet - Medium-chain triglyceride (MCT) supplementation in long-chain defects (VLCAD, LCHAD)
Disorders of Carbohydrate Metabolism	- Classic Galactosemia (GALT) - Galactokinase Deficiency (GALK) - Epimerase Deficiency (GALE)	Milk feeding intolerance Prolonged jaundice Vomiting, diarrhea Hepatomegaly Liver failure	- Elevated bilirubin (direct and indirect) - Increased serum galactose - Generalized aminoaciduria - Low urinary osmolality	Within the first 48 h with the start of milk feeding	- Immediate discontinuation of lactose-containing milk - Galactose-free diet
Lysosomal Storage Disorders with Neonatal Onset	- Mucopolysaccharidosis Type I (MPS I, Hurler Syndrome) - Pompe Disease - GM1 Gangliosidosis - Niemann-Pick Disease Type A	Severe hypotonia Cardiomyopathy (Pompe) Hepatomegaly Splenomegaly Dysmorphisms	- Increased tissue glycogen (Pompe) - Enzyme deficiency (enzymatic assay) - Elevated urinary glycosaminoglycans (MPS I)	First weeks of life, but rarely in the first days	- Enzyme replacement therapy (ERT) for treatable conditions (Pompe, MPS I) - Bone marrow transplantation in selected cases
Disorders of Cysteine and Homocysteine Metabolism	- Homocystinuria (CBS) - Cystinuria - Nephropathic Cystinosis	Lethargy, feeding difficulties Growth delay Progressive renal impairment (cystinosis)	- Elevated plasma homocysteine levels (homocystinuria) - Lysosomal cystine accumulation (cystinosis)	Weeks to months of life, but may begin in the first days	- Low-methionine diet and betaine (homocystinuria) - Cysteamine therapy (cystinosis)

**Table 2:** Overview of the six reported cases.

Patient ID (Sex)	Age at Publication	Diagnosis (Disease Category)	Genetic Confirmation	Clinical Onset (Timing)	Ammonium Peak (µmol/L)	Treatment	Resolution	Outcome/ Sequelae	Transplant
p1 (M)	6.5 y.o.	Cit I (UCD)	ASS gene: c.1168G > A/ c.1168G > A	Lethargy, dyspnea, convulsions (2nd day of life)	770	Detoxification, Peritoneal dialysis	6 days	Epileptic encephalopathy	No
p2 (F)	N.A. *	Cit I (UCD)	ASS gene: c.905T > G/ c.1030C > T	Hyporeactivity, dyspnea, metabolic acidosis (2nd day of life)	1218	Detoxification, Peritoneal dialysis	N.A.	Deceased (19th day of life)	N.A.
p3 (M)	N.A.	ASA (UCD)	ASL gene: c.1129C > G/ c.1322G > A	Lethargy, coma (4th day of life)	1381	Detoxification, Peritoneal dialysis	N.A.	Deceased (11th day of life)	N.A.
p4 (M)	1.5 y.o.	MMA_mut0 (OA)	MUT gene: c.785G > A/ c.2104_2197 delGCCGinsTGGAA	Lethargy, dyspnea, metabolic acidosis (3rd day of life)	849	Detoxification, Continuous Veno-Venous Hemofiltration (CVVH)	6 days	None	Yes
p5 (M)	11.5 y.o.	MMA_mut0 (OA)	MUT gene: c.1207C > T/ c.1207C > T	Tachypnea, hypotonia, hypovolemic shock, severe acidosis (3rd day of life)	1100	Detoxification, Peritoneal dialysis	6 days	Severe metabolic encephalopathy	Yes
p6 (F)	13 months	MCADD (FAODD)	ACADM gene: c.997A > G/ c.997A > G	Hyporeactivity, hypoglycemia, metabolic acidosis, heart failure (2nd day of life)	N.A.	Carnitine, Inotropes, High-flow IV dextrose	1 week	None	N.A.

* N.A. = Not Applicable.

## Data Availability

All clinical data and materials are available in our Pediatric Unit.

## Abbreviations

The following abbreviations are used in this manuscript:

ENS	Expanded Newborn Screening
IMDs	Inherited Metabolic Diseases
UCDs	Urea Cycle Disorders
CIT1	Citrullinemia type 1
ASA	Argininosuccinic Acidemia
MMA	Methylmalonic Acidemia
MCADD	Medium-Chain Acyl-CoA Dehydrogenase Deficiency
DBS	Dried Blood Spot
OAs	Organic Acidurias
FAODs	Fatty Acid Oxidation Disorders
MS/MS	Tandem Mass Spectrometry
2-TT	Second Tier Test
NICU	Neonatal Intensive Care Units
IV	Intravenous
